# Experimental Investigation and Theoretical Modelling of a High-Pressure Pneumatic Catapult Considering Dynamic Leakage and Convection

**DOI:** 10.3390/e22091010

**Published:** 2020-09-10

**Authors:** Jie Ren, Jianlin Zhong, Lin Yao, Zhongwei Guan

**Affiliations:** 1 School of Mechanical Engineering, Nanjing University of Science and Technology, Nanjing 210094, China; zhongjianlin@njust.edu.cn; 2 School of Engineering, University of Liverpool, Liverpool L69 3GQ, UK; Zhongwei.Guan@liverpool.ac.uk; 3 Nanjing Research Institute on Simulation Technique, Nanjing 210016, China; yaolinxueshu@163.com

**Keywords:** thermodynamics, mathematical modelling, dynamic leakage, convective heat transfer, compressibility factor, pneumatic catapult

## Abstract

A high-pressure pneumatic catapult works under extreme boundaries such as high-pressure and rapid change of pressure and temperature, with the features of nonlinearity and gas-solid convection. In the thermodynamics processes, the pressure is much larger than the critical pressure, and the compressibility factor can deviate from the Zeno line significantly. Therefore, the pneumatic performance and thermo-physical properties need to be described with the real gas hypothesis instead of the ideal gas one. It is found that the analytical results based on the ideal gas model overestimate the performance of the catapult, in comparison to the test data. To obtain a theoretical model with dynamic leakage compensation, leakage tests are carried out, and the relationship among the leakage rate, pressure and stroke is fitted. The compressibility factor library of the equation of state for compressed air is established and evaluated by referring it to the Nelson-Obert generalized compressibility charts. Based on the Peng–Robinson equation, a theoretical model of the high-pressure pneumatic catapult is developed, in which the effects of dynamic leakage and the forced convective heat transfer between the gas and the metal wall are taken into account. The results from the theoretical model are consistent with the data from ejection tests. This research presents an approach to study the performance of a high-pressure pneumatic catapult with high precision.

## 1. Introduction

High-pressure pneumatic catapult works with high-pressure air, which has the advantages of no pollution, inexpensive nature, recycling, high power density and stable performance [1]. On top of high-pressure, it is also characterized by high-speed and heavy load. It is widely adopted in the industrial field of automation and robot driving. It can also be applied to aerospace and marine [2,3,4].

A high-pressure pneumatic catapult has the characteristics of high nonlinearity, rapid change of gas-solid convective heat transfer and strong real gas effects. In thermodynamics processes, the pressure is much higher than the critical pressure, and the compressibility factors deviate from the Zeno line significantly. In this situation, the analytical results from an ideal gas model have a large deviation; furthermore, as a result of this, the accuracy of the theoretical model based on the ideal gas hypothesis is not suitable for theoretical analysis and practical implementation [5,6]. Therefore, the pneumatic performance and thermo-physical properties need to be described with the real gas hypothesis. However, some previous studies are based on the hypothesis of the ideal gas and adiabatic, and some are considered on the real gas effects without considering the convective heat transfer [7,8,9]. Few studies take the real gas effects and the convective heat transfer into consideration at the same time. Furthermore, the research on the theoretical modelling of a high-pressure catapult with a consideration of dynamic leakage and convective heat transfer is rarely reported, nor is the experimental work on the dynamic leakage of a catapult. Here, a novel high-pressure pneumatic catapult with an open-type cylinder is adopted to drive a heavy object with a high velocity, in which the compressed air with strong instantaneous expansibility is chosen for the pneumatic catapult [10,11]. Accurate theoretical modelling of a high-pressure pneumatic catapult is a prerequisite condition for the design and implementation of such systems. Many equations have been proposed to describe the properties of real gases, including the van der Waals equation [12,13], the virial equation [14], the Redlich–Kwong equation [15], the Benedict–Webb–Rubin equation [16], the Soave–Redlich–Kwong equation [17], and the Peng–Robinson equation of state [18,19], which are applicable to gases within a certain pressure and temperature range.

To date, most of the studies on theoretical modelling of pneumatic catapult have been based on the assumption of the ideal gas and adiabatic or based on real gas assumptions, but without considering convective heat transfer and dynamic leakage, with few researchers taking the real gas effects, convective heat transfer, and leakage into account all in one theoretical model. Besides, the accuracy of theoretical models is not fully evaluated by experimental work. In this study, ejection tests of an experimental catapult prototype were first carried out, in which test results were used to compare with the theoretical model of the ideal gas state. Then, leakage tests of the pneumatic catapult were carried out to obtain a precise model for dynamic leakage, by fitting the relationship among leakage rate, pressure and stroke. Finally, by considering the dynamic leakage and the forced convective heat transfer between the working medium and the metal wall in the real gas state, an accurate theoretical model of a high-pressure pneumatic catapult was established, which was verified by the experimental data. This research provides support for designing and optimizing high-pressure pneumatic catapults.

## 2. Experimental Study on Ejection of a High-Pressure Pneumatic Catapult

### 2.1. The Working Mechanism of a High-Pressure Pneumatic Catapult

**Figure 1:**
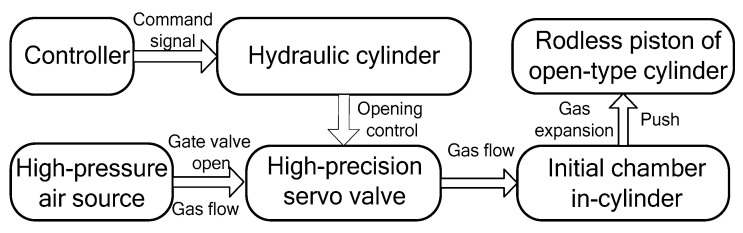
A workflow chart of the high-pressure pneumatic catapult.

Figure 1 shows a workflow chart of a high-pressure pneumatic catapult with an open-type cylinder, which is comprised of a controller, servo valve-controlled module (including a hydraulic cylinder and a high-precision servo valve), an open-type cylinder, and a high-pressure air source. The servo valve-controlled module is used for setting the movement of the valve core for the throttle control of high-pressure air to meet pressure change. The working mechanism of the high-pressure pneumatic catapult can be described as follows, i.e., the servo valve-controlled module starts to work with the control command, and the gas flows into the chamber of an open-type cylinder from the gas source after opening the high-precision servo valve. Then, the acceleration of analogue load attached to the piston is executed by the high-pressure gas in the cylinder. When ejection is finished, the gas in the cylinder is relieved and the velocity of the piston is reduced to zero by a buffering cylinder.

**Figure 2:**
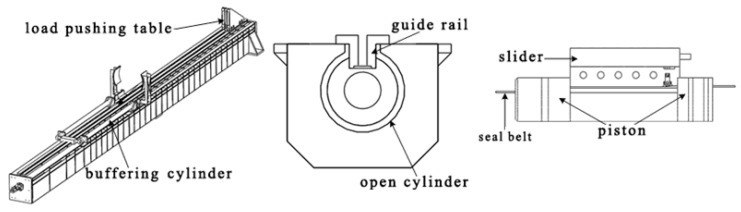
Schematic of an open-type cylinder.

The open-type cylinder, shown in Figure 2, is a key component in the high-pressure pneumatic catapult, which mainly includes an open cylinder, piston, seal belt, guide rail, load pushing table and buffering cylinder. The load pushing table is a loading platform, which is attached to piston for transferring movement. The buffering cylinder is designed to provide a shelter for the load pushing table and piston. A set of holes are set to relieve the pressure in the cylinder when ejection is completed.

### 2.2. The Ejection Test System of a High-Pressure Pneumatic Catapult

**Figure 3:**
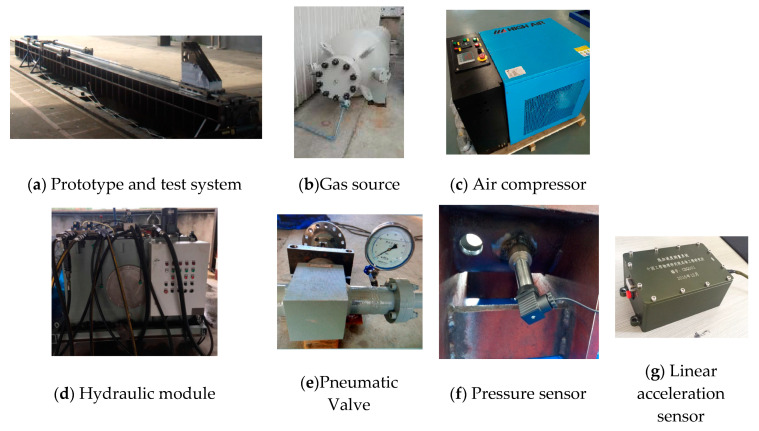

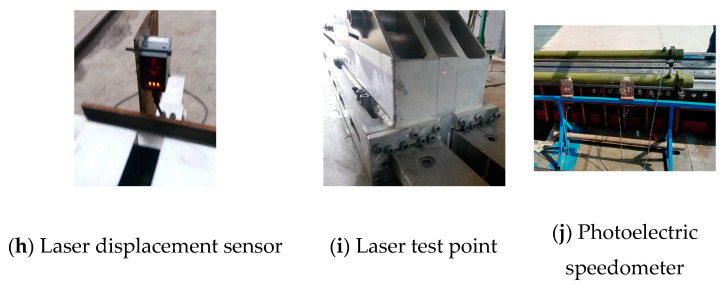
The ejection test system of a high-pressure pneumatic catapult.

The ejection test system of a high-pressure pneumatic catapult is shown in Figure 3. Figure 3a is a prototype and test system. Figure 3b–e show the gas source, air compressor, hydraulic module, and pneumatic valve, respectively. Pressure sensors with 0.075% F. S. accuracy and 50 MPa range are installed on the cylinder to measure the pressure, and a wireless linear acceleration sensor with 20 g range and voltage sensitivity 100 mv/g is used to measure the ejection acceleration, as shown in Figure 3f,g. Figure 3h,i show the laser displacement sensor with 50 m range and ± 0.12% F. S. accuracy tracked the piston displacement during the ejection. Two photoelectric sensors with 450 mm range and 0.5 ms accuracy are used in the terminal velocity subsystem to record the instantaneous velocity at the end of the stroke, as shown in Figure 3j.

### 2.3. Comparison of the Ejection Testing and the Theoretical Model in the Ideal Gas State

Ejection studies were carried out based on the catapult prototype, with the test results being compared with the analytical results of the theoretical model in the ideal gas state, and the accuracy of the ideal gas model being assessed.

**Figure 4:**
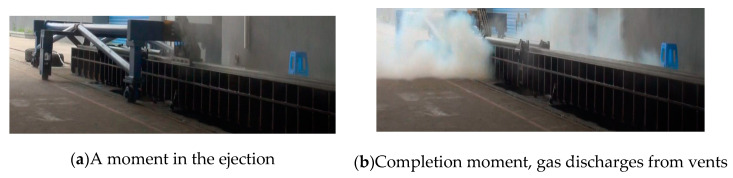
Ejection test of the high-pressure pneumatic catapult at 30 MPa.

**Figure 5:**
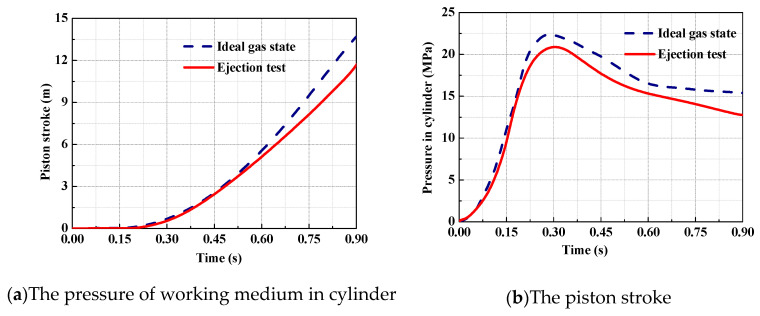
Contrasts of the test results and analytical results of the ideal gas model.

Here, an ejection test case is shown in Figure 4. In this example, the initial pressure of the gas source is set to 30 MPa; Figure 4a shows a catapult moment in the ejection and Figure 4b shows the gas discharges from vents. Comparing the analytical results in the ideal gas state with the test results under the same conditions, the contrasts are shown in Figure 5. It is found that, without considering the leakage and convective heat transfer, the maximum deviation on the piston stroke and the gas pressure in the cylinder are 16.7 and 24.5%, respectively. Moreover, the trend of the deviation is getting bigger with time. As a result, the precision of the ideal gas model is unacceptable for practical engineering applications.

## 3. Experimental Investigation Leakage of the High-Pressure Pneumatic Catapult

A high-pressure pneumatic catapult is equipped with an open-type cylinder, which indicates that there will be leakage. The leakage rate is closely linked to the gas flow and pressure in the cylinder, which affects the accuracy of the theoretical model of the pneumatic catapult [20,21,22]. As the piston moves fast during the ejection, the leakage area increases accordingly, so that leakage rate changes significantly. The dynamic model of the leakage rate and coefficients can be fitted by leakage testing.

### 3.1. Schematic Diagram of the Test System on Leakage Rate

**Figure 6:**
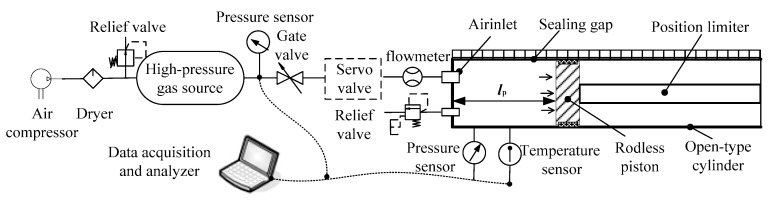
Schematic diagram of leakage tests.

Figure 6 shows a schematic diagram of a leakage testing system, which consists of an open-type cylinder, position limiter, high-pressure gas source, servo valve, gate valve, relief valve, pressure sensors, temperature sensor, air compressor, dryer, and data acquisition. In this work, the leakage measurement adopts positive pressure detection method, by taking several fixed positions within the stroke of the piston, loading different pressures respectively, as well as measuring and recording the data of gas flow, pressure and temperature. The state parameters in the gas source and the cylinder are measured in a relatively stable state, which can be applied to the ideal gas law. With these test data, the leakages and leakage rate of the working medium during the valve opening process can be inferred.

### 3.2. The Principle of Leakage Rate Test and Test Procedure

In order to build the leakage rate model of high-pressure air in the ejection process, it is necessary to estimate the leakages with different strokes and pressures. In leakage tests, there is a large internal force between the piston and the cylinder due to the piston being constrained by the position limiter. Maintaining a high-pressure for a long time may cause structural deformation. To guarantee safety, various low pressures are set for the leakage test. For the floating seal structure in an open-type cylinder, the concept of design is that the greater the pressure, the smaller the seal gap, and the better the sealing performance. Therefore, the leakage in a low pressure is greater than that in a high pressure. A testing pressure up to 4 MPa can be applied to evaluate the outer envelope of the leakage rate, with an unexaggerated performance of the system. In addition, if necessary, the correlation between leakage and pressure difference can be further calibrated by the relationship between leakage and pressure [23,24,25].

The procedures of the leaking test are as follows. Firstly, adjust the piston in the specified positions with a limiter. Secondly, supplement high-pressure air for the gas source using an air compressor. Thirdly, open the gate valve and the pneumatic servo valve to allow the high-pressure air to flow into the open cylinder from the gas source, and keep the valve opening until the system reaches the steady-state. Finally, record the pressure changes of the gas in the gas source and open-type cylinder by the data acquisition, then close the valve and relieve the pressure. To make the data reliable, the measurements were repeated three times and an average value was taken.

In these tests, the piston positions were set to six different values, i.e., 0, 1.1, 2.2, 3.3, 4.2, and 5.2 m. Similarly, the pressures of working medium in-cylinder were set as 2.0, 2.75, and 3.8 MPa. Keep the valve open for about 10 s for retaining enough time to complete the test. The density and the remaining mass of the working medium can be calculated from the measured pressure and temperature after opening the valve according to the standard atmospheric conditions, and the leakage can be obtained.

### 3.3. Leakage Tests and Fitting on Dynamic Leakage Rate Model

**Figure 7:**
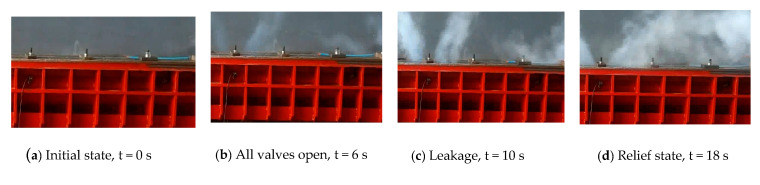
An example of leakage test at maximum pressure 3.8 MPa in cylinder.

**Figure 8:**
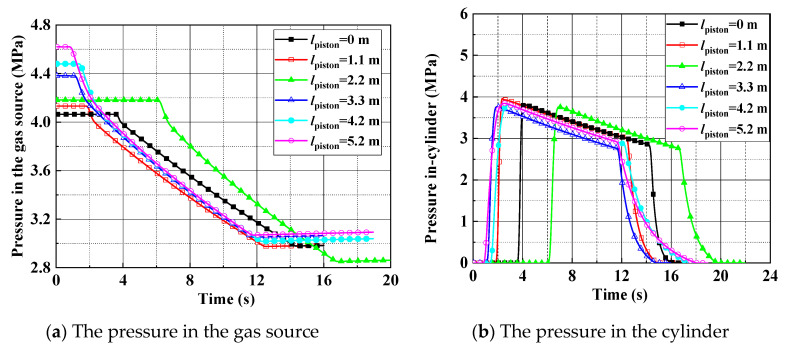
Test data of the pressure at the maximum pressure 3.8 MPa in cylinder.

**Figure 9:**
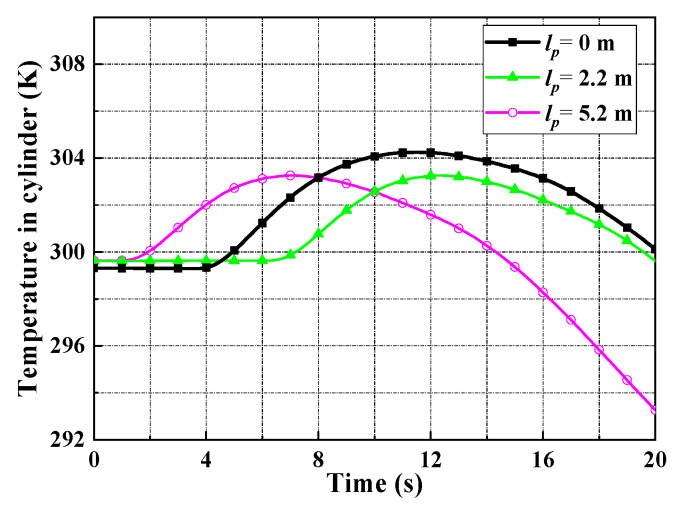
The temperature at the maximum pressure 3.8 MPa in cylinder.

A leak test is shown in Figure 7; in this test case, the pressure sensor with 8 MPa range and 0.25% F. S. accuracy and the temperature sensor with 0.2% F. S. accuracy and −50 °C–150 °C range. The pressure of a working medium in the gas source is 4.2 MPa, the piston fixed position is 2.2 m, and the pressure of relief valve is set to 4.0 MPa to guarantee safety. At the time of 6 s, all valves are fully opened and kept open for about 10 s. The pressure in cylinder reaches a maximum soon after the high-pressure airflow into the cylinder. Due to the leakage, the pressure drops after reaches the peak, as shown in Figure 8. The high-pressure air leaked forms a white mist, which can be seen in Figure 7. From Figure 7a,b, it can be seen that the leakage occurs when all valves open, then leakage is increasing, as shown in Figure 7c. The open-type cylinder cannot maintain high-pressure for a long time, as it may be damaged and cause an accident. Therefore, the leakage duration is maintained about 10 s to meet the test requirements, then the servo valve is closed, while the relief valve is open, as shown in Figure 7d. Taking the test with 3.8 MPa pressure in cylinder as a representative case, the test data of the pressure and temperature are shown in Figure 8 and Figure 9. With these test data, the leakage rate can be inferred from the equation of state for a real gas, as shown in Table 1.

**Figure 10:**
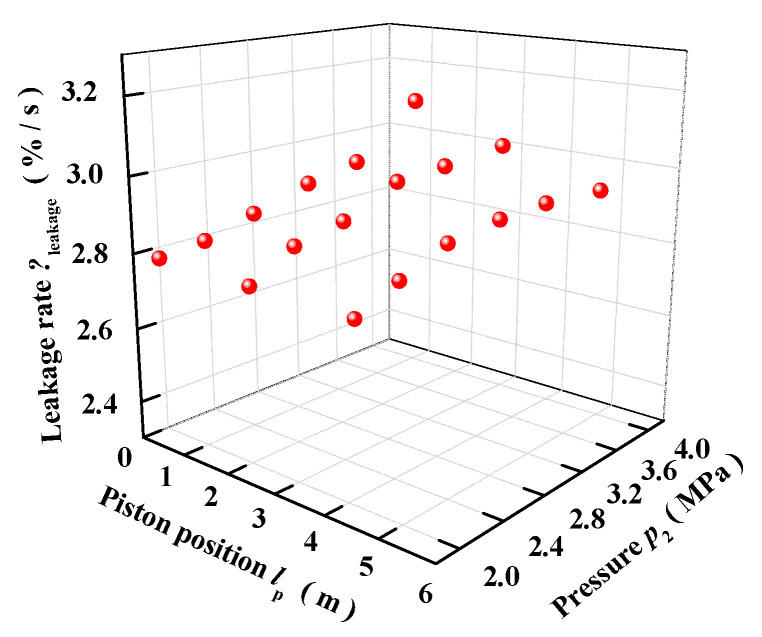
The leakage rate.

According to Table 1 and Figure 10, the least-squares method is used to fit the dynamic leakage rate model, which is a quadratic one. The relationship of the leakage rate *η*_leak_ and the pressure and piston position is fitted as follows.

(1) $$\eta_{\mathrm{leak}}=k_{1}+kp_{2}+k_{3}lp+k_{4}p_{2}lp+k_{5}p_{2}^{2}+k_{6}l_{p}^{2}$$

where *k*_1_, *k*_2_, *k*_3_, *k*_4_, *k*_5_, and *k*_6_ are the fitted coefficients of the leakage rate model. For this catapult, the coefficients with 95% confidence bounds are *k*_1_ = 2.864 × 10^−2^, *k*_2_ = 9.549 × 10^−4^, *k*_3_ =2.202 × 10^−5^, *k*_4_ = −4.465 × 10^−5^, *k*_5_ = 9.998 × 10^−5^, and *k*_6_ = −3.228 × 10^−4^, respectively.

## 4. Modelling of the High-Pressure Pneumatic Catapult Based on Real Gas Consideration

### 4.1. The Theoretical Modelling Flow of the High-Pressure Pneumatic Catapult

**Figure 11:**
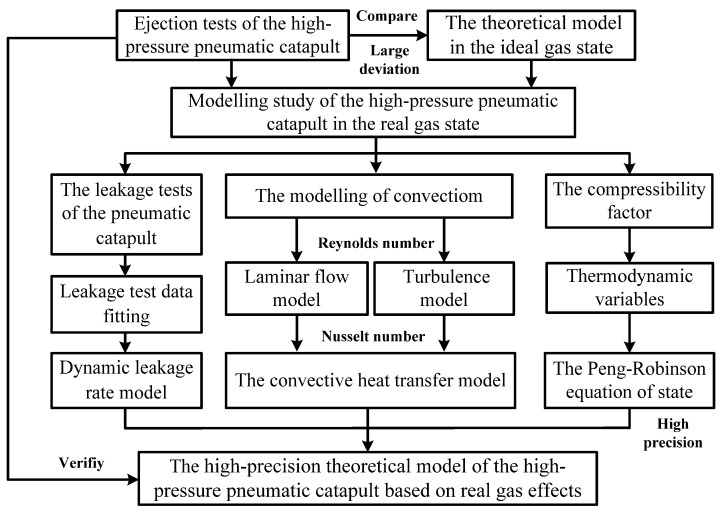
A modeling flow chart of the high-pressure pneumatic catapult.

A flow chart for modelling the high-pressure pneumatic catapult is given in Figure 11. Based on the Peng–Robinson equation of state for real gases, by considering the effects of dynamic leakage and taking into account the convective heat transfer between the working medium and the metal wall, a high-precision theoretical model of the high-pressure pneumatic catapult is developed. The simulated transient gas pressure and temperature are verified with the test data of the ejection test of the high-pressure pneumatic catapult.

### 4.2. The Convective Heat Transfer between the Working Medium and the Metal Wall

For the working medium inside a high-pressure gas source, such a source is a large heat one with high heat capacity. It can be assumed that the temperature of the gas source body stays constant during work. Therefore, for the gas source, the heat convection between the outer surface of the gas source and external air can be ignored, and only that between the working medium and the inner wall surface of the gas source needs to be considered, as does that in the cylinder.

The convective heat transfer includes forced convection heat one and natural convection heat one. When the catapult ejects, the working medium flows into the cylinder from the gas source, which is generated by the external force. Thus, it is the forced convection heat transfer between the working medium and the metal wall, and the heat flux can be calculated by Newton’s law of cooling.

When the working medium is heated, the rate of heat increase can be expressed as below.

(2) $$\frac{dQ}{\mathrm{dt}}=\alpha A\Delta T\left(t\right)=hA\left(T_{w}-T_{f}\right)$$

When the working medium is cooled, the rate of heat loss is shown as follows.

(3) $$\frac{dQ}{\mathrm{dt}}=\alpha A\Delta T\left(t\right)=\alpha A\left(T_{f}-T_{w}\right)$$

where *α* is the heat transfer coefficient, *Q* the heat, *A* the heat transfer surface area, *T_w_* and *T_f_* are the temperature of the wall and working medium respectively.

When Reynolds number Re ≤ 2300, the convective heat transfer is a laminar heat transfer, and the Nusselt number is calculated by the Sieder–Tate equation.

(4) Nuf=1.86(Ref⋅Pr⋅dL)1/3(ηfηw)0.14

where Pr is the Prandtl number, *d* the characteristic inner diameter of the flow channel, *L* the characteristic length, *η_f_* and *η_w_* are the viscosity when the qualitative temperature is the temperature of the working meduim and the wall. If the relationship between gas viscosity and temperature is not considered, *η_f_*/*η_w_* = 1.

When Re > 2300, it is a turbulent heat transfer, and the Nusselt number needs to be calculated by the Dittus–Boelter correction equation.

(5) Nuf=0.023Ref0.8⋅Prf0.4⋅εl⋅εR⋅εt

where $\varepsilon_{l}$, $\varepsilon_{R}$ and $\varepsilon_{t}$ are the tube length, bending and temperature correction factor, respectively.

When the ejection starts, all the valves are fully opened, the working medium flows into the cylinder, and the pressure and the temperature of the working medium rise rapidly in a small enclosed space. Then the piston is pushed to move fast, while the internal volume of the cylinder increases, resulting in continually decreasing the temperature and the pressure of the working medium. The gas source is in the state of deflation and the temperature of the working medium in the gas source decreases further. Consequently, the working medium is heated by the inner wall surface of the gas source.

According to Newton’s law of cooling [12], the heat flux between the working medium and the inner wall of the gas source is as follows.

(6) δQ1=α1A1(Tw−Tf)={1.86(Ref⋅Prf⋅dL)1/3(ηfηw)0.14⋅λD1⋅A1(Tw1−Tf1),Ref≤23000.023Ref0.8⋅Prf0.4⋅εl⋅εR⋅εt⋅λD1⋅A1(Tw1−Tf1),Ref>2300

and the heat flux between the working medium and the inner wall surface of the cylinder is

(7) δQ2=α2A2(Tf−Tw)={1.86(Ref⋅Prf⋅dL)1/3(ηfηw)0.14⋅λD2⋅A2(Tf2−Tw2),Ref≤23000.023Ref0.8⋅Prf0.3⋅εl⋅εR⋅εt⋅λD2⋅A2(Tf2−Tw2),Ref>2300

The heat transfer coefficient *α* can be calculated by Nusselt number, as shown in Equation (8).

(8) $$\alpha =Nu_{f}\lambda /d$$

where *λ* is the thermal conductivity of fluid, *d* the characteristic inner diameter of round tube.

### 4.3. Compressibility Factor and Thermodynamic Variables of a Real Gas

#### 4.3.1. Compressibility Factor of a Real Gas

Compressibility factor is a function of the corresponding pressure and temperature, and it can be used as a basis to select the equation of state. As the working medium is dry air, the virial equation, Soave–Redlich–Kwong equation and Peng–Robinson (P-R) equation are all applicable to the high-pressure pneumatic system. From our previous research, the P-R equation of state is more suitable for an open-type catapult. The expression of the compressibility factor is shown as follows.

(9) $$Z=\frac{pV_{m}}{RT}$$

where *Z* is the compressibility factor, *V*_m_ the molar volume, *R* the universal gas constant, *p* the pressure, *T* the temperature.

The P-R equation is an improvement on the van der Waals equation, as follows.

(10) $$p=\frac{RT}{V_{m}-b}-\frac{a(T)}{V_{m}(V_{m}+b)+b(V_{m}-b)}$$

where *a*(*T*) and *b* are van der Waals constants.

The cubic equation for the molar volume *V*_m_ can be obtained after solve the P-R equation of state. Together with Equations (9) and (10), the compression factor at a special point can be obtained.

(11) $$Z=(\frac{RT}{V_{m}-b}-\frac{a(T)}{V_{m}(V_{m}+b)+b(V_{m}-b)})V_{m}/RT$$

For dry air, when the pressure is less than half of the critical pressure or the temperature is greater than 5 times of the critical temperature, the *P*-*Z* curves can be approximately considered as linear and the compressibility factor *Z* is nearly 1. When the pressure is greater than half of the critical or the temperature is less than 5 times of the critical temperature, a reliable real gas equation of state needs to be derived to fit the data. The Nelson-Obert (N-O) generalized compressibility charts proposed by Nelson-Obert are the most precise ones, with pressures of *p*_r_ = 0~1 MPa (meiobar range), *p*_r_ = 1~10 MPa (middle-pressure range) and *p*_r_ = 10~100 MPa (high-pressure range).

From the ideal gas model, it can be known that the temperature range of gas in a cylinder is from 220 K to 463.5 K. Although it is larger than the actual range, it still can be a basis for selection of temperature. The comparison of compressibility factors for air under the specified pressure and temperature with the N-O compressibility charts and P-R equation of state is shown in Table 2.

As noted in Table 2, when the pressure is relatively low, compressibility factors are close to 1.0 within the temperature range of the table. The deviation of the compressibility factors corresponding to the P-R equation and N-O values is small, which means the ideal gas model can be applied when the pressure is low. With the increase of the pressure and temperature, the compressibility factor gradually deviates from 1.0. The difference in pressure and temperature between real gas and ideal gas cannot be neglected in such situations. Within the changing range of temperature and pressure of the ejection, the P-R equation has a high accuracy.

#### 4.3.2. Thermodynamic Variables

The real gas thermodynamic property variables can be obtained by subtracting the residual function from the ideal value. The definition of residual function can be expressed as

*F*_re_ = F* − *F* (12)

where *F*_re_ denotes the residual of a arbitrary extensive properties or specific properties, F* and *F* are the properties of ideal gas and real gas.

The residual specific thermodynamic energy for real gas is as follows.

(13) $$u_{r}=\int_{\infty }^{v}\left[p-T(\frac{\partial p}{\partial T})\right]dv=-T^{2}{\int_{\infty }^{v}\left[\frac{\partial (p/T)}{\partial T}\right]}_{v}dv$$

The specific enthalpy for real gas and ideal gas are defined as

*h* = *u* + *pv* (14)

(15) $$h^{*}=u^{*}+R_{g}T$$

where *h* is the specific enthalpy, *u* specific thermodynamic energy, *R_g_* is gas constant, *p* the pressure, *T* the temperature, superscripts * denotes ideal gas.

Combination of Equations (12)–(15) will lead to

(16) $$h_{r}=-T^{2}{\int_{\infty }^{v}\left[\frac{\partial (p/T)}{\partial T}\right]}_{v}dv-pv+R_{g}T$$

Therefore, by introducing the P-R equation, the expressions of the specific thermodynamic energy can be derived as follows.

(17) $$u=C_{v}T-2.0778R_{g}T_{c}\left[-{\left(r+1\right)}^{2}+\frac{\left(r^{2}+r\right)}{T_{c}{}^{0.5}}T^{0.5}\right]\cdot \ln\left[\frac{V_{m}+0.0778\left(1-\sqrt{2}\right)\frac{RT_{c}}{P_{c}}}{V_{m}+0.0778\left(1+\sqrt{2}\right)\frac{RT_{c}}{P_{c}}}\right]$$

where *R* is the Molar gas constant, *T_c_* the critical temperature, *P_c_* the critical pressure, *V*_m_ the molar volume, *v* the specific volume and *v* *= V*_m_/*M*, *r* the characteristic constant determined by material types, *C_v_* the specific heat at constant volume of ideal gas state.

The experimental results show that the specific heat capacity of an ideal gas is a complex function of temperature, which increases with temperature. The formula of air is in a cubic relationship between the specific constant pressure heat capacity *C_p_* and temperature [26,27]. By combining with the Meyer formula, *C_p_* can be obtained by the equation below.

(18) $$C_{p}=C_{v}+R_{g}=C_{0}+C_{1}\theta +C_{2}\theta^{2}+C_{3}\theta^{3}$$

where $\theta ={\left(T\right)}_{k}/1000$. To dry air, there are $C_{0}=1.05$, $C_{1}=-0.365$, $C_{2}=0.85$, $C_{3}=-0.39$.

### 4.4. The Accurate Theoretical Model Considering Dynamic Leakage and Convective Heat Transfer

In the procedure of ejection, the mass flow equation of the pneumatic valve can be written as

(19) $$G_{c}=\left\{ \begin{array}{l} \mu_{x}\frac{p_{1}A_{v}}{\sqrt{R_{g}T_{1}}}\sqrt{\frac{2k}{k-1}\left[{\left(\frac{p_{2}}{p_{1}}\right)}^{\frac{2}{k}}-{\left(\frac{p_{2}}{p_{1}}\right)}^{\frac{k+1}{k}}\right]},\;{\left(\frac{2}{k+1}\right)}^{\frac{k}{k-1}}<\frac{p_{2}}{p_{1}}<1\\ \mu_{x}\sqrt{k}{\left(\frac{2}{k+1}\right)}^{\frac{k+1}{2(k-1)}}p_{1}A_{v}/\sqrt{R_{g}T_{c}},\;\frac{p_{2}}{p_{1}}\leq {\left(\frac{2}{k+1}\right)}^{\frac{k}{k-1}} \end{array}\right.$$

where *G*_c_ is the mass flow at the throttle, subscripts 1 and 2 indicate gas source and cylinder, respectively, *μ*_x_ the flow correction factor, *A*_v_ the equivalent flow area of a large flow pneumatic valve, *k* the adiabatic index, *R*_g_ the gas constant, *T*_c_ the critical temperature of a working medium, *T*_1_ the working temperature of a working medium in gas source, *p*_1_ and *p*_2_ the pressures of working medium in gas source and cylinder.

The mass equation and the energy equation of a working medium for a high-pressure gas source can be expressed in Equations (20) and (21) below, respectively.

(20) $$\frac{d}{dt}\left(\rho_{1}V_{1}\right)=-G_{c}$$

(21) $$\frac{d}{dt}\left(\rho_{1}V_{1}u_{1}\right)=-G_{c}h_{1}$$

The equation for the motion of a piston can be expressed as:

(22) $$\frac{dv_{p}}{dt}=\frac{p_{2}S-(m_{a}+m_{t})g-f}{\left(m_{a}+m_{t}\right)}$$

where *v*_p_ is the velocity of piston, *S* the effective pushing area of piston, *g* the acceleration of gravity, *f* is the friction force, *m_a_* the mass of analogue load, *m_t_* the total mass of piston and load pushing table, *p*_2_ the pressure of working medium in cylinder.

The energy equation of a working medium for the cylinder is shown.

(23) $$\frac{d\left(m_{2}u_{2}\right)}{dt}=G_{c}h_{1}-Sp_{2}\frac{dl_{p}}{dt}$$

where *m*_2_ is the mass of the working medium in the cylinder.

The pressure and temperature in the cylinder rise firstly and then decrease, which vary greatly during the ejection. Based on the conservation laws of mass and energy, mass and flow equations, P-R state equations, coupled dynamic leakage models and convective heat transfer models, an accurate theoretical model of the high-pressure pneumatic catapult is established and can be expressed as the closed-form equations. There are seven independent variables, i.e., *ρ*_1_, *T*_1_, *m*_2_, *T*_2_, *l*_p_, *v*_p_ and *A*_v_. Let *X*_1_ = *ρ*_1_, *X*_2_ = *T*_1_, *X*_3_ = *m*_2_, *X*_4_ = *T*_2_, *X*_5_ = *l*_p_, *X*_6_ = *v*_p_ and *X*_7_ = *A*_v_. The closed-form equations can be calculated by five-stage fourth-order Runge–Kutta method.

(24) $$\left\{ \begin{array}{l} {\dot{X}}_{1}V_{1}+G_{c}=0\\ {\dot{X}}_{2}W_{1}-{\dot{X}}_{1}\cdot Y_{1}-\frac{{\dot{X}}_{1}V_{1}\left(h_{1}-u_{1}\right)u_{1}+\delta Q_{1}}{X_{1}}=0\\ {\dot{X}}_{3}-G_{c}\cdot \eta_{\mathrm{leak}}=0\\ {\dot{X}}_{4}X_{3}W_{2}+\left[G_{c}u_{2}+S_{2}p_{2}X_{6}-0.85G_{c}h_{1}-Y_{2}\cdot \frac{MS_{2}X_{3}X_{6}-M\left(V_{01}+S_{2}X_{5}\right){\dot{X}}_{3}}{X_{3}}+\delta Q_{2}\right]=0\\ {\dot{X}}_{5}-X_{6}=0\\ {\dot{X}}_{6}-\frac{S_{2}p_{2}-(m_{a}+m_{t})g-f}{\left(m_{a}+m_{t}\right)}=0 \end{array}\right.$$

where $u_{1}=C_{v_{1}}X_{2}-Y_{1}$, $u_{2}=C_{v2}X_{4}-Y_{2}$, $h_{1}=u_{1}+\frac{p_{1}}{X_{1}}$,

$$\eta_{\mathrm{leak}}=k_{1}+k_{2}p_{2}+k_{3}X_{5}+k_{4}p_{2}X_{5}+k_{5}p_{2}^{2}+k_{6}X_{5}^{2}$$

$$C_{v_{1}}=C_{0}+C_{1}\frac{X_{2}}{1000}+C_{2}{\left(\frac{X_{2}}{1000}\right)}^{2}+C_{3}{\left(\frac{X_{2}}{1000}\right)}^{3}-R_{g},$$

$$C_{v_{2}}=C_{0}+C_{1}\frac{X_{4}}{1000}+C_{2}{\left(\frac{X_{4}}{1000}\right)}^{2}+C_{3}{\left(\frac{X_{4}}{1000}\right)}^{3}-R_{g}$$

$$G_{c}=\left\{ \begin{array}{l} \mu_{x}\frac{p_{1}X_{7}}{\sqrt{R_{g}T_{1}}}\sqrt{\frac{2k}{k-1}\left[{\left(\frac{p_{2}}{p_{1}}\right)}^{\frac{2}{k}}-{\left(\frac{p_{2}}{p_{1}}\right)}^{\frac{k+1}{k}}\right]}{\left(\frac{2}{k+1}\right)}^{\frac{k}{k-1}}<\frac{p_{2}}{p_{1}}<1\\ \mu_{x}\sqrt{k}{\left(\frac{2}{k+1}\right)}^{\frac{k+1}{2(k-1)}}p_{1}X_{7}/\sqrt{R_{g}T_{c}}\;\frac{p_{2}}{p_{1}}\leq {\left(\frac{2}{k+1}\right)}^{\frac{k}{k-1}} \end{array}\right.$$

$$W_{1}=\left[\left(C_{0}-\frac{C_{1}}{10^{3}}\cdot 2X_{2}+\frac{C_{2}}{10^{6}}\cdot 3X_{2}^{2}+\frac{C_{3}}{10^{9}}\cdot 4X_{2}^{3}-R_{g}\right)-1.0389R_{g}T_{c}^{0.5}(r^{2}+r)X_{2}^{-0.5}\ln\left(\frac{V_{m1}+0.0778(1-\sqrt{2})\frac{RT_{c}}{p_{c}}}{V_{m1}+0.0778(1+\sqrt{2})\frac{RT_{c}}{p_{c}}}\right)\right]$$

$$W_{2}=\left[\left(C_{0}-\frac{C_{1}}{10^{3}}\cdot 2X_{4}+\frac{C_{2}}{10^{6}}\cdot 3X_{4}^{2}+\frac{C_{3}}{10^{9}}\cdot 4X_{4}^{3}-R_{g}\right)-1.0389R_{g}T_{c}\frac{\left(r^{2}+r\right)}{T_{c}^{0.5}}X_{4}^{-0.5}\ln\left(\frac{V_{m2}+0.0778(1-\sqrt{2})\frac{RT_{c}}{p_{c}}}{V_{m2}+0.0778(1+\sqrt{2})\frac{RT_{c}}{p_{c}}}\right)\right]$$

$$Y_{1}=2.0778R_{g}T_{c}[-{\left(r+1\right)}^{2}+\frac{\left(r^{2}+r\right)}{T_{c}^{0.5}}X_{2}^{0.5}]\cdot \left(\frac{1}{V_{m1}+0.0778(1-\sqrt{2})\frac{RT_{c}}{p_{c}}}-\frac{1}{V_{m1}+0.0778(1+\sqrt{2})\frac{RT_{c}}{p_{c}}}\right)$$

$$Y_{2}=2.0778R_{g}T_{c}[-{\left(r+1\right)}^{2}+\frac{\left(r^{2}+r\right)}{T_{c}^{0.5}}X_{4}^{0.5}]\cdot \left(\frac{1}{V_{m2}+0.0778(1-\sqrt{2})\frac{RT_{c}}{p_{c}}}-\frac{1}{V_{m2}+0.0778(1+\sqrt{2})\frac{RT_{c}}{p_{c}}}\right)$$

## 5. Verification of the Accuracy of the Theoretical Model with the Ejection Test

### 5.1. Verification of the Accuracy of the Theoretical Model Based on Real Gas Effects

**Figure 12:**
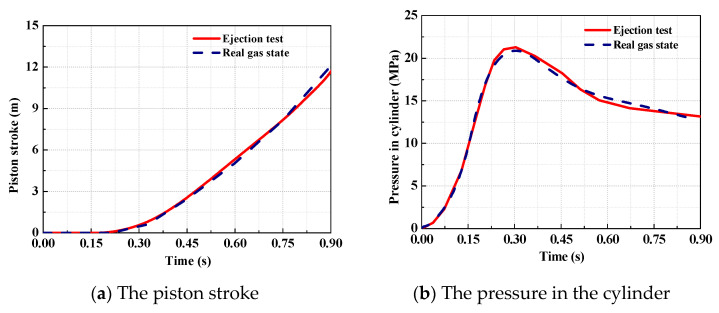
The analytical results and test data of the pressure in the cylinder.

Comparing the analytical results in the real gas state with the data from ejection test can help one understand how reliable the model is. Based on the theoretical model developed, an analysis program is worked out. Then, the analytical results of the ejection based on the real gas effects are obtained, which are compared with the test data of the ejection obtained in Section 3.2, as shown in Figure 12.

As indicated in Figure 12, it can be seen that the analytical results are in good agreement with the test data. When the initial pressure of working medium in the gas source is set to 30 MPa, the maximum deviation of ejection stroke between the analytical results and the test data is 3.7%, and the maximum pressure deviation of working medium in the cylinder is 4%. It shows that the theoretical model of high-pressure pneumatic catapult developed in the real gas state has a high precision.

### 5.2. Comparison of Theoretical Models of High-Pressure Pneumatic Catapult

**Figure 13:**
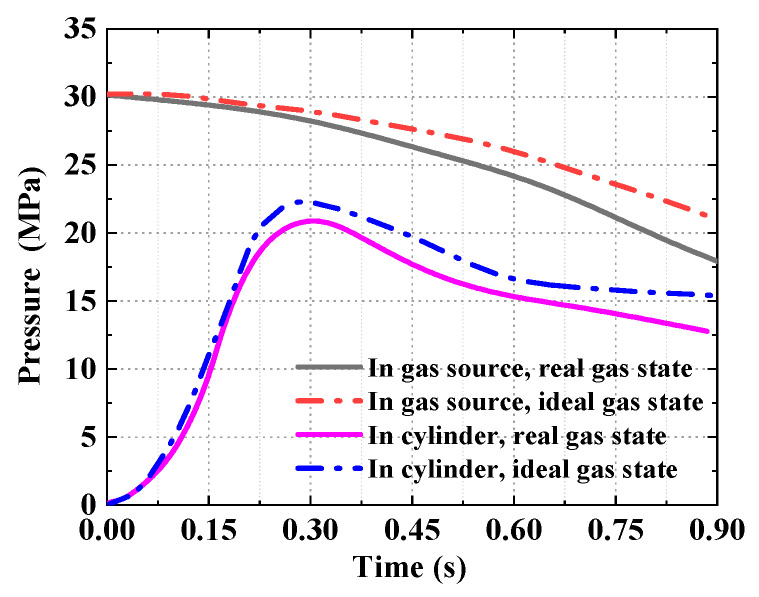
The analytical results of pressure.

**Figure 14:**
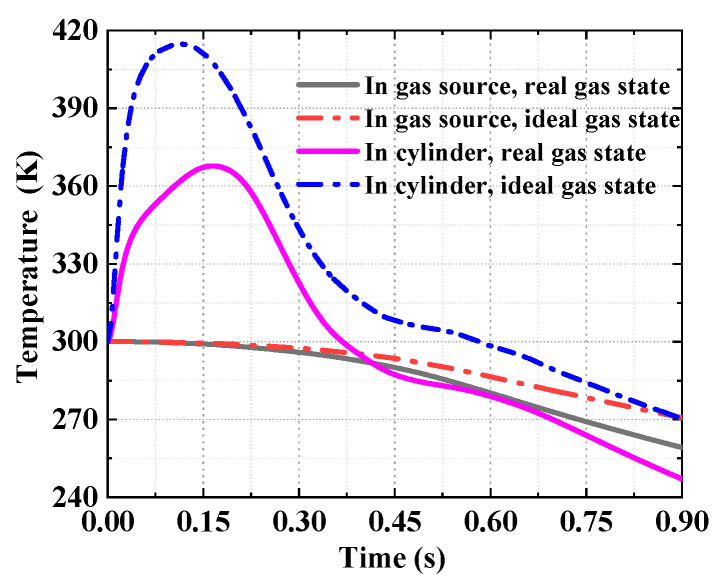
The analytical results of temperature.

**Figure 15:**
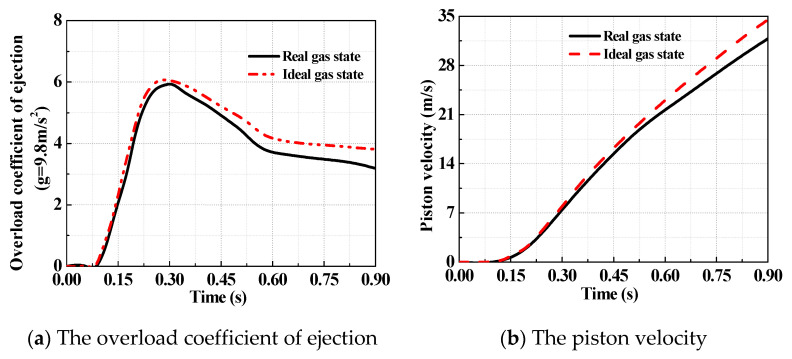
The analytical results of ejection performance parameters.

The comparison of analytical results for the ejection in the ideal gas state and the real gas state are shown in Figure 13, Figure 14 and Figure 15. Figure 13 shows the analytical results of the pressure in the gas source and cylinder. It can be seen that the analytical results in the ideal gas state are clearly larger than that in the real gas state, with the maximum deviation of 22%, while the initial parameters of the gas source are the same. Figure 14 shows the temperature of the working medium in the gas source and cylinder. Whether to consider dynamic leakage and convective heat transfer or not, the temperature deviation of working medium reached 12% in the cylinder, and the maximum temperature difference is 55.4 K, which indicates the effects of dynamic leakage and convective heat transfer cannot be ignored. Moreover, Figure 15 shows the deviation of the ejection overload coefficient and the piston velocity reaches 20 and 12%, respectively.

All the values related to the ideal gas state are higher than those to the real gas state. In the initial stage, the deviation is small and however, it becomes increasingly large. This means that the ability of the high-pressure pneumatic catapult in the ideal gas state is exaggerated. Therefore, a high-precision assessment of pneumatic catapult requires taking the effects of dynamic leakage and convective heat transfer into account in the theoretical model, in order to gain the necessary accuracy of the model.

## 6. Conclusions

Based on the experimental and theoretical work of the high-pressure pneumatic catapult presented, the following conclusions can be drawn.

(1) It is found that the analytical results based on the ideal gas model give an overestimated performance of the catapult, in comparison to the test data. The maximum deviation of the piston stroke and the cylinder gas pressure is 16.7% and 24.5%. Consequently, the precision of the ideal gas model is unacceptable for the engineering applications.
(2) The relationship of the leakage rate, pressure and stroke is fitted. It is found that the maximum leakage rate of the pneumatic catapult does not exceed 5% within the whole piston stroke, showing a good sealing performance. The leakage rate model is a key factor that affects the accuracy of the theoretical model. Taking leakage into account can improve the accuracy of the theoretical model and make the theoretical calculation more consistent with the actual situation. Regardless of the leakage, the theoretical model is not a true high precision model, and cannot be used to evaluate actual pneumatic catapults, and its practicality will be limited.
(3) A corresponding convective heat transfer model between the working medium and the metal wall has been developed. In the heat transfer process, the choice of laminar flow model or turbulent heat transfer model is based on the Reynolds number.
(4) Based on the Peng–Robinson equation, a theoretical model of the high-pressure pneumatic catapult has been developed, in which the effects of dynamic leakage and the forced convective heat transfer between the gas and the metal wall are taken into account. The results from the theoretical model are consistent with the data form ejection tests, with a maximum deviation of 4%, indicating much higher precision than the ideal gas model.
(5) The theoretical model established can be applicable for dry gases, such as an air, N_2_ and CO_2_. It cannot be applied to catapults that use water vapor as it involves phase change of working medium in ejection. The equation of state is not applicable to the two-phase regions.

## Figures and Tables

**Table 1:** The leakage rates at different pressure and positions.

The Leakage Rate *η*_leakage_ [%/s]		Piston Position *l*_p_ [m]					
		0.00	1.10	2.20	3.30	4.20	5.20
Maximum pressure in-cylinder *p*_2_ [MPa]	2.00	2.77	2.85	2.95	3.05	3.12	3.27
	2.75	2.62	2.77	2.87	3.00	3.06	3.13
	3.80	2.41	2.57	2.72	2.82	2.89	2.95

**Table 2:** Compressibility factors of air under the specified pressure and temperature.

Compressibility Factors *Z*			Pressure [MPa]							
			0.101	1	6	12	18	24	30	35
Temperature [K]	220 (*T_r_* = 1.6)	P-R equation	0.9982	0.9793	0.8960	0.8521	0.8647	0.9322	1.0969	1.1259
		N-O value	0.9981	0.9938	0.9068	0.8421	0.8666	0.9425	1.0388	1.1310
	265 (*T_r_* = 2)	P-R equation	0.9990	0.9903	0.9550	0.9421	0.9555	0.9903	1.0326	1.0762
		N-O value	0.9988	0.9928	0.9683	0.9588	0.9761	1.0039	1.0152	1.0276
	331 (*T_r_* = 2.5)	P-R equation	0.9997	0.9975	0.9920	0.9989	1.0323	1.0564	1.0914	1.1137
		N-O value	0.9969	0.9975	0.9996	1.0101	1.0429	1.0753	1.1034	1.1328
	463.5 (*T_r_* = 3.5)	P-R equation	1.00027	1.0038	1.0146	1.0336	1.0662	1.0929	1.1086	1.1321
		N-O value	1.0033	1.0042	1.0181	1.0405	1.0709	1.1067	1.1236	1.1527
